# Age‐Specific Survival Estimation of a Eurasian Crane Population Highlights a Long‐Term Decline in Juvenile Survival

**DOI:** 10.1002/ece3.72779

**Published:** 2026-02-03

**Authors:** Morgane Gicquel, Juan C. Alonso, Lovisa Nilsson, Matthew Low, Javier A. Alonso, Dmitrijs Boiko, Damon Bridge, Patrick Dulau, Thomas Heinicke, Anne Kettner, Yosef Kiat, Petras Kurlavičius, Sigvard Lundgren, Michael Modrow, Günter Nowald, Ivar Ojaste, Alain Salvi, Jostein Sandvik, Markéta Ticháčková, Antonio Torrijo, Jari Valkama, Zsolt Végvári, Johan Månsson

**Affiliations:** ^1^ Grimsö Wildlife Research Station, Department of Ecology Swedish University of Agricultural Sciences Riddarhyttan Sweden; ^2^ Department of Evolutionary Ecology, Museo Nacional de Ciencias Naturales Consejo Superior de Investigaciones Científicas (CSIC), José Gutiérrez Abascal 2 Madrid Spain; ^3^ Department of Ecology Swedish University of Agricultural Sciences Uppsala Sweden; ^4^ Department of Biodiversity, Ecology and Evolution, Faculty of Biological Sciences, Universidad Complutense, José Antonio Novais 12 Madrid Spain; ^5^ Latvian National Museum of Natural History Rīga Latvia; ^6^ The Faculty of Medicine and Life Sciences of the University of Latvia, Jelgavas Iela 1 Rīga Latvia; ^7^ RSPB West Sedgemoor Reserve Office, Dewland's Farm, Langport, Somerset Somerset UK; ^8^ Species Survival Commission – Crane Specialist Group IUCN Gland Switzerland; ^9^ Kranichschutz Deutschland NABU‐Kranichzentrum Groß Mohrdorf Germany; ^10^ School of Zoology, Faculty of Life Sciences Tel Aviv University Tel Aviv Israel; ^11^ Vytautas Magnus University Kaunas Lithuania; ^12^ Swedish Crane Working Group Älvshult, Smeagården Ambjörnarp Sweden; ^13^ Institute of Agricultural and Environmental Sciences Estonian University of Life Sciences Tartu Estonia; ^14^ Conservatoire D'espaces Naturels de Lorraine France; ^15^ Selbu Norway; ^16^ Crane Life CZ, Liberec Zoo Liberec Czech Republic; ^17^ Amigos de Gallocanta Zaragoza Spain; ^18^ Ringing Centre, Finnish Museum of Natural History University of Helsinki Helsinki Finland; ^19^ Institute of Aquatic Ecology Hun‐Ren Centre for Ecological Research Budapest Hungary; ^20^ Senckenberg Deutsches Entomologisches Institut Müncheberg Germany

**Keywords:** bird banding, capture‐recapture, *Grus grus*, life expectancy, sex‐specific, survival rates

## Abstract

The Eurasian crane ( *Grus grus* ), a symbol of conservation success in Europe, has made an impressive recovery since the 1979 Birds Directive, with current estimates reaching around 590,000 individuals. However, this transition from vulnerability to abundance brings new challenges, particularly arising from interactions with human activities (e.g., conflicts with agriculture). Understanding the future dynamics of crane populations requires knowledge of demographic parameters that are crucial for predicting population trends and informing management and conservation measures. We analysed 37 years of data (1985–2021) from 5049 juvenile‐banded cranes and 172,725 resightings, providing estimates of survival rates across age classes and over time. Our findings indicate that juveniles exhibit the lowest survival rates, while sub‐adults have higher survival, and adults show a decrease in their survival probability with age, as expected with the senescence process. Over the study period, juvenile survival declined by almost 30% overall, while sub‐adults experienced a smaller decrease, and adults showed no change. Life expectancy at birth was 10 years, and maximum lifespan reached 25 years. We found no difference in the survival estimates of males and females. The decline in juvenile survival over the years highlights the growing challenges likely driven by habitat degradation, climate change, agricultural practices, and increasing population densities. These findings align with previous research on crane survival and underscore the importance of understanding age‐specific survival dynamics in response to environmental changes. This study highlights the challenges facing Eurasian crane populations, where further declines in juvenile and immature survival rates could lead to population declines unless compensated by a stable or higher adult survival. Effective conservation strategies will require further research into reproductive success and details on age‐specific mortality causes and environmental pressures, although targeted interventions can already be implemented to mitigate current impacts of habitat degradation and climate change.

## Graphical abstract

The Eurasian crane (*Grus grus*), a symbol of conservation success in Europe, has made an impressive recovery. However, our results show that over the study period, juvenile survival declined by almost 30% overall, while sub‐adults experienced a smaller decrease, and adults showed no change. The decline in juvenile survival over the years highlights increasing challenges likely driven by habitat degradation, climate change, agricultural practices, and rising population densities.

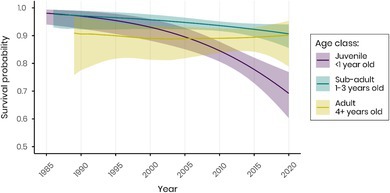

## 1 Introduction

Survival is a key demographic parameter related to species' population dynamics (Newton 1998), and thus its estimation is an important component of wildlife conservation and management programmes (Sandercock 2006). Understanding variation in age‐ or life‐stage‐specific survival is necessary for predicting population trends, identifying vulnerable life stages, and determining the population‐regulating factors driving this variation (Bonenfant et al. 2009; Low and Pärt 2009). This information is essential for developing effective population management strategies, such as determining which life‐stage survival rates have the greatest impact on population growth (Coulson et al. 2005) and therefore where and when specific management interventions should be targeted (Low et al. 2010). Long‐term survival trends clarify population trajectories and identify life stages that benefit most from management or show resilience to environmental and anthropogenic pressures.

Migratory waterbirds represent a diverse group for which both negative and positive survival and population trends have been observed in relation to land use changes (Fox and Madsen 2017; Hemminger et al. 2022). Negative impacts may arise from wetland loss and degradation (Ma et al. 2023; Verhoeven and Setter 2010), climate change impacts on food resources and nesting sites within wetlands (Amano et al. 2020), and disease outbreaks (e.g., avian influenza: AIV‐H5N1) linked to higher bird concentrations at increasingly limited staging sites (European Food Safety Authority et al. 2023; Miller 2022). However, some species that are able to exploit agricultural resources may react positively to land use changes, leading to rapid population growth and high concentrations of birds that increasingly bring them into conflict with farmers and present new challenges to their conservation management (Fox and Madsen 2017). One example is the Eurasian crane ( *Grus grus* ; hereafter crane), whose populations have undergone extensive growth since the 1980s as a result of conservation efforts and the expansion and intensification of agriculture in their European wintering areas (Alonso et al. 2016; Ilyashenko 2016; Mewes 1994; Prange and Ilyashenko 2019).

The crane's ability to thrive in agricultural landscapes in combination with restrictions on its hunting resulted in its IUCN classification being updated from ‘vulnerable’ to ‘least concern’ in 2015 (BirdLife International 2016). The magnitude of its population growth is illustrated by comparing the number of individuals that passed through Gallocanta lake, the main staging area of the western flyway, during the spring migration of 1985 (31,945 individuals) (Alonso, Alonso, and Cantos 1986) with those of recent years (165,949 individuals, average of the last six spring migrations 2020–2025; J. A. Román, 2020–2025). A similar increase was predicted by a flow model analysing the total migratory passage population of this species at Gallocanta between 1985 and 2015 (Drever et al. 2025). The current population estimate of Eurasian cranes is over 590,000 individuals (Nowald 2024; Prange and Ilyashenko 2019). While this recovery represents a significant conservation success, it has led to increased human‐bird conflicts (Alonso et al. 2018; Nilsson et al. 2019), resulting from crop damage and yield losses for farmers, especially on farmland near wetlands where these birds concentrate in high numbers (Nilsson et al. 2016, 2019). This issue has escalated in Europe due to the increased number of cranes at staging and wintering areas, along with prolonged staging periods (Alonso et al. 1994; Montràs‐Janer et al. 2020; Nowald et al. 2018; Salvi 2010). Additionally, cranes face risks from collisions with aircraft, power lines, and wind turbines, presenting challenges for authorities and infrastructure managers (Fanke et al. 2011).

In the context of the ongoing expansion of this crane population, there is a clear need to understand its demographic rates. This will be the first time survival values in this population have been estimated since the beginning of the population's recovery (Alonso et al. 1991; Alonso, Quintanilla, and López 1986). Therefore, this study is aiming to provide one of the baseline demographic data required to understand population growth and inform management strategies for sustainable population control, if these become necessary. To do so, we used a large collaborative database encompassing 5049 cranes banded in 11 European countries with over 170,000 resightings, ranging from 1985 to 2021, and we estimated age‐dependent survival rates and their changes over several decades, and from this calculated important parameters such as maximum lifespan and life expectancy for different ages.

## 2 Methods

### 2.1 Study Species

The crane is a large migratory bird, and its European population has a breeding range spanning northern and eastern Europe (Prange 2005; Prange and Ilyashenko 2019). During winter, it migrates to southern latitudes, primarily in southern Europe, northern Africa and sometimes reaching central Africa (Alonso et al. 2016; Hafid et al. 2010; Salvi 2016a, 2016b; Spina et al. 2022). European cranes travel along three primary migration routes: the Western, Central or Baltic‐Hungarian, and Eastern flyways (Leito et al. 2015). Cranes demonstrate high site fidelity, often returning to the same breeding, staging, and wintering areas annually (J. A. Alonso et al. 2008; Alonso et al. 2004; Nowald 2001; Román Álvarez 2024). They begin reproducing at around 4 years of age (Alonso and Alonso 1999; Archibald et al. 2020; Barwisch et al. 2022). During the breeding season, cranes primarily inhabit boreal wetlands, peat bogs, and open forested landscapes to establish territorial breeding sites (Cramp and Simmons 1980), where they lay one or two eggs (Leito et al. 2005). They have also adapted to nesting in agricultural lands and near human settlements. Cranes are omnivorous, their diet being primarily plants, such as rhizomes, roots, leaves, berries, seeds, cereals, but also invertebrates, and occasionally amphibians, reptiles, rodents and birds (Hemminger et al. 2022; Nowald et al. 2018; Nowald and Fleckstein 2001).

### 2.2 Crane Banding and Data Collection

Most cranes were hand‐captured at their natal areas at 6–8 weeks old using a short‐distance run from a vehicle or a hide (see Månsson et al. 2013). A few juveniles were captured in their wintering range, in either Spain or France (respectively 11 and two individuals), when they were moulting (Alonso et al. 2018). For those juveniles, natal origin was determined if they were subsequently observed within the breeding range during next breeding seasons. Moulting occurs in adult cranes every 2 years, typically during the summer, and cranes are therefore unable to fly for approximately 5 weeks (Glutz et al. 1973; Prange 1989). Each crane was marked with either an alphanumeric ring (in the first year of the initiative) or a unique combination of coloured plastic rings on one or both tibiotarsi, in addition to the national scheme metal ring on the tarsometatarsus. Since 1988, country codes with three coloured plastic bands on the left tibia and individual codes with three bands on the right tibia were used. Seven different colours of plastic rings were available: white, yellow, red, blue, green, black, and brown. Marked juveniles were released immediately after banding. The coloured bands enable readings and identification of individuals from a distance using telescopes or cameras. Since the start of crane banding in 1985, several types of bands have been used (Alonso et al. 2018; Gicquel et al. 2025; Prange and Ilyashenko 2019). As the species is monomorphic in plumage, and shows overall low sexual dimorphism, sex was only identified when pair mating events were witnessed, or through non‐systematic molecular sexing techniques (Alonso et al. 2019). Individual information on when or how sex was determined was not available, so sex was treated as a fixed attribute for all individuals identified as male or female.

### 2.3 Data Processing

This study relies on banding and resighting data collected across several European countries, with the majority of resightings reported by birdwatchers during opportunistic observations distributed across the main breeding, staging and wintering areas of each flyway. National coordinators review each resighting event and check its consistency with valid and existing colour‐band combinations before inclusion in the national or iCORA database (internet‐based Crane Observation Ring Archive, Crane Conservation Germany 2009; Heinicke et al. 2016). From the combination of all datasets, we compiled information for all cranes on each of their banding and resighting events, with the respective date, location (address, place), and geographical coordinates (longitude and latitude). All records containing any detectable errors and inaccuracies were omitted (e.g., observations of unknown colour‐band combinations, uncertain crane identity). The curated dataset yielded a total of 5049 individual cranes that were banded as juveniles in 11 different countries across Europe (Germany, Finland, Sweden, Norway, Latvia, Estonia, Lithuania, United Kingdom, Czech Republic, France and Spain), and with known natal origin (i.e., natal country) and 172,725 resightings, between 1985 and 2021 (Table A.1 in Appendix S1). Data from cranes hatched and ringed in the framework of a crane reintroduction project in the United Kingdom were included. Of 5049 banded cranes included in models, 995 (20%) were never resighted after banding (0%–25% depending on country, Table A.1 in Appendix S1).

### 2.4 Modelling

We converted the raw banding and resighting dataset into annual capture and recapture sequences for each individual crane, using calendar years as temporal units, resulting in 37 annual occasions during the study period (1985–2021). We removed cranes that were banded the last year (in 2021).

As a first step, we tested the goodness of fit (GOF) of the data using the R package *R2ucare* (Gimenez et al. 2018), a R version of the software U‐CARE (Choquet et al. 2009). The overall dataset presented a considerable lack of fit, with tests for trap dependence (Test 2.CT), transience (Test 3.SR) and overdispersion (Tests 2.CL and 3.SM) all being significant (Table B.1 in Appendix S1). We therefore fitted models using the mixture extension ‘CJSMixture’ of the Cormack–Jolly–Seber ‘CJS’ model using the *RMark* package (Laake 2013) in the software R (v. 4.3.1, R Core Team 2023), combined with program MARK (White and Burnham 1999), to estimate the survival probability *ϕ* and the detection probability *p* of cranes. Although resightings were collected opportunistically throughout the year, focusing on annual survival and using mixture models likely minimises any bias from the violation of the instantaneous sampling assumption. Mixture models are known to be an efficient way to account for heterogeneity of undetermined origin and using two classes is generally considered sufficient (Pledger et al. 2003).

The survival parameter (*ϕ*) was modelled using age, time and natal origin, with:

1. age: modelled by three life stages: juvenile (0 year old), subadult (1–3 years old), and adult (4+ years old). Juvenile is implicitly included in the model as the reference category (intercept). To allow within‐group age effects, we incorporated: Subadult and Adult as categorical (binary) variables (0/1, indicating presence in the respective age class). Age is a continuous variable capturing the linear effects of age within each life stage. This approach allows for distinct survival estimates for juveniles while maintaining a linear and gradual change in survival rates within the sub‐adult and adult age classes,
2. time (T): following a temporal linear trend,
3. natal country: assuming a difference in survival rates for cranes of different origin, reflecting different environmental effects, and also accounting for differences arising from the use of different migratory flyways.

Detection probability (*p*) was modelled as a function of biologically and methodologically relevant covariates that could influence the likelihood of detecting an individual. The factors considered were:

1. colourband: categorical variable to account for differences in detection probability based on the type of bands used, as they may vary in visibility and readability,
2. time since marking (tsm): fitting a linear trend accounting for decrease in detection rates arising from degradation of colourbands,
3. time (T): fitting a linear temporal trend of detection probability to account for increasing observation over the years,
4. natal country: accounting for a potential variation in detection due to observation effort variation from different countries and migratory flyways,
5. mixture: accounting for unexplained heterogeneity in detection probability among individuals by dividing individuals into two latent detection groups (high detectability vs. low detectability)

The mixture parameter pi was kept constant, meaning that the proportion of the population belonging to each mixture group is fixed rather than a time or other covariate dependent process.

Given the period and extent of use of each band type, as well as the start of banding schemes in different countries, specific parameters were fixed to prevent estimating parameters that were realistically not possible (see Table A.2 in Appendix S1). To account for overdispersion, which can bias more complex models by favouring complexity and narrowing confidence intervals, the Variance Inflation Factor (Fletcher ĉ, calculated from the most general model: 8.58) was adjusted using the adjust.chat function in RMark. We performed model‐averaging of the two top‐ranked models (ΔQAICc ≤ 7; Burnham et al. 2011) after ĉ adjustment (Table C.1 in Appendix S1). Confidence intervals in the text and figures reflect the adjustment and model averaging process. We also corrected model ranking using an alternative VIF metric, the median ĉ (1.45). We tested median ĉ as a robustness check because Fletcher ĉ can overestimate over dispersion when data are sparse. It led to a different model being selected as the top‐ranked one after ĉ adjustment (see Supporting Information D in Appendix S1). However, the beta estimates and predictions from this model (see Tables D.1 and D.2 and Figures D.1–3 in Appendix S1) were similar to the ones obtained from our model averaging process, showing that our results are robust. As parameter estimates were highly similar under both approaches, all reported results are based on Fletcher ĉ adjustment.

We also fitted another model on a subset of the data, with cranes that have been sexed (*N*_female_ = 1089, *N*_male_ = 1288). We used the two highest‐ranked models identified as described above and added the effect of sex in interaction with other variables on the survival parameter *ϕ* (Table E.1). We adjusted models (with Fletcher ĉ = 10.75) and performed a model averaging of those two equivalent models.

### 2.5 Calculation of Maximum Lifespan and Life Expectancy

#### 2.5.1 Maximum Lifespan

**FIGURE 1:**
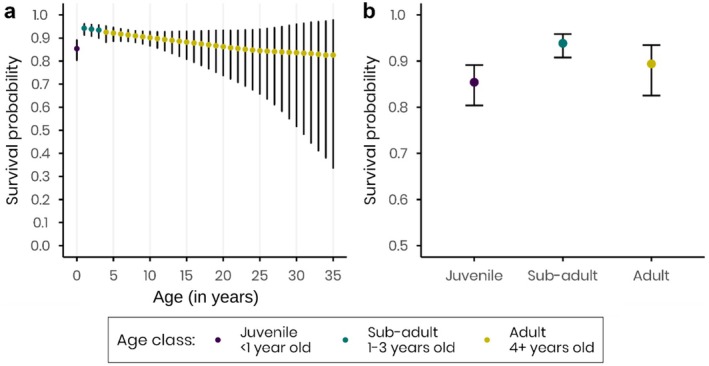
Survival probabilities of banded European cranes, between 1985 and 2021. (a) Age‐specific survival, and (b) average survival probability per age‐class, derived by model averaging. The colours represent three different age classes, purple: Juveniles (less than 1 year old), green: Sub‐adults (1–3 years old) and yellow: Adults (4+ years old).

Maximum lifespan is the greatest age reached by any individual of a species, representing the biological limit for survival of that species. To calculate it, we followed the definition and method presented by Mayne et al. (2020). We estimated the cumulative survival across ages based on the survival probabilities (𝜙) obtained from the CMR model (see Figure 1b), with estimates of their lower and upper 95% confidence intervals. Cumulative survival at each age was calculated as the product of the survival probabilities up to that age:

$$S\left(x\right)=\prod_{i=1}^{x}\phi_{i}$$

where *S*(𝑥) is the cumulative survival probability at age 𝑥. Confidence intervals for cumulative survival were calculated with the same formula, using the lower and upper confidence limits of the 𝜙 values.

Then, maximum lifespan (m_95%_) was defined as the age at which the cumulative survival reached below 5%, i.e., *S*(𝑥) < 0.05.

#### 2.5.2 Life Expectancy

Life expectancy is the average number of years an individual is expected to live from a specific age, and was calculated using the formula:

$$e_{x}=\frac{\sum_{i=x}^{m}l_{i}}{l_{x}}$$

where 𝑒_𝑥_ is the life expectancy at age 𝑥, 𝑙_𝑖_ is the survival probability at age 𝑖, and 𝑚 is the maximum age (m_95%_). The sum $\sum_{i=x}^{m}l_{i}$ was computed over the remaining survival probabilities from age 𝑥 up to the maximum age (m_95%_). Confidence intervals for life expectancy were calculated in the same manner, by applying the formula to the lower and upper confidence limits of 𝑙_𝑖_.

## 3 Results

Following our model adjustment based on Fletcher correction and our model selection, the averaged model included the variables coding for age‐classes (Age + Subadult + Adult), as well as a temporal trend (T) and their interaction on the apparent survival parameter. For the detection parameter, colourband type, time since marking (tsm), temporal trend (T), natal country and mixture group were selected, with one of the best models including also the interaction between colourband and tsm (Table C.1 in Appendix S1). Two mixture groups were identified: one accounting for 45% of the cranes of the dataset with an estimated detection probability of 0.30, and another accounting for the remaining 55% of the population, with an estimated detection probability of 0.72. Results on detection probability are available in a previous study (Gicquel et al. 2025).

### 3.1 Age‐Specific Survival

Juvenile cranes showed the lowest survival rates of all age classes considered (estimated mean and 95% CI: 0.85 [0.80, 0.89]), adults showed an intermediate survival (0.89 [0.82, 0.93]), and sub‐adults showed the highest survival (0.94 [0.91, 0.96], Figure 1a,b). When considering individual survival as a linear function of age (excluding juvenile class), the probability of surviving decreased by 0.38% each year on average (Figure 1b).

### 3.2 Temporal Trends in Survival

**FIGURE 2:**
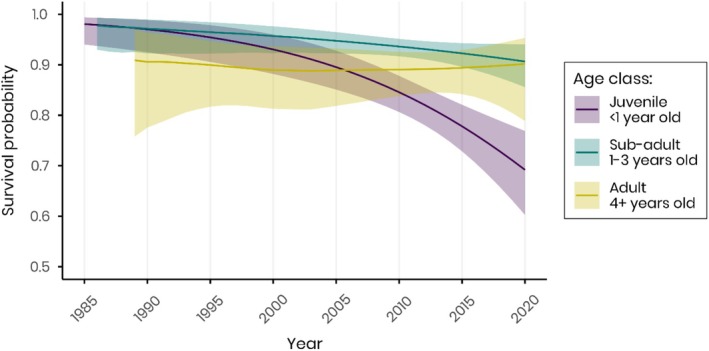
Survival probabilities of banded European cranes for specific age‐class over the years (temporal trend *T*), between 1985 and 2021, derived by model averaging. The colours represent three different age stages, purple: Juveniles (less than 1 year old), green: Sub‐adults (1–3 years old) and yellow: Adults (4+ years old).

The survival of juveniles has been decreasing by on average 1% annually over the study period, with this decline accelerating over time (Table 1, Figure 2). Over the 37 years of the study, juvenile survival probability decreased from 0.98 in 1985 to 0.69 in 2020 (i.e., a change in overall survival of 29.59%). In sub‐adults, there was a smaller decrease in survival of 0.22% per year, and overall a decline of 7.07%. In contrast, adults exhibited relatively stable survival probabilities over time, with only minor fluctuations and no significant long‐term decline (0.32% overall, odd‐ratio ≈1).

**TABLE 1:** Parameter beta estimates of the two best fitting models (see Table C.1 in Appendix S1) for estimating survival and detection probability of banded cranes between 1985 and 2021. Respective QAICc weight for model 1 and model 2 are 0.55 and 0.45. Key variables include age classes: juveniles (intercept), sub‐adults, adults; time since marking (*tsm*) in years, temporal trend (*T*), natal country and band types (colourband), for parameters (Par.) of mixture (pi), survival (Phi) and detection (p). LCI and UCI respectively represent lower and upper 95% confidence intervals.

Par.	Variable	Model 1				Model 2			
		Est.	SE	LCI	UCI	Est.	SE	LCI	UCI
pi	Intercept	−0.18	0.06	−0.29	−0.07	−0.20	0.06	−0.31	−0.09
Phi	Intercept	3.92	0.21	3.52	4.32	3.94	0.21	3.54	4.35
	Age	−0.10	0.05	−0.21	0.01	−0.12	0.05	−0.22	−0.02
	Subadult	0.03	0.32	−0.61	0.67	−0.01	0.33	−0.65	0.63
	Adult	−1.18	0.47	−2.11	−0.25	−1.30	0.47	−2.22	−0.38
	*T*	−0.09	0.01	−0.10	−0.08	−0.09	0.01	−0.10	−0.08
	Age: *T*	0.002	0.002	−0.002	0.01	0.002	0.002	−0.001	0.01
	Subadult: *T*	0.04	0.01	0.02	0.07	0.04	0.01	0.02	0.07
	Adult: *T*	0.09	0.02	0.06	0.12	0.10	0.02	0.06	0.13
p	Intercept	−2.32	0.39	−3.07	−1.56	−3.07	0.40	−3.87	−2.28
	Colourband—Finnish	1.74	0.18	1.38	2.09	2.22	0.23	1.78	2.67
	Colourband—Spanish	1.04	0.19	0.66	1.42	1.91	0.23	1.45	2.36
	Colourband—ELSA	1.10	0.21	0.69	1.50	1.79	0.25	1.30	2.27
	*tsm*	−0.21	0.01	−0.22	−0.19	−0.07	0.03	−0.12	−0.03
	*T*	0.09	0.01	0.08	0.10	0.10	0.01	0.08	0.11
	Natal Country—Estonia	−1.44	0.35	−2.13	−0.75	−1.45	0.35	−2.14	−0.76
	Natal Country—Finland	−1.66	0.36	−2.37	−0.95	−1.63	0.36	−2.34	−0.92
	Natal Country—Germany	0.43	0.35	−0.25	1.11	0.43	0.35	−0.25	1.11
	Natal Country—Latvia	−0.54	0.50	−1.51	0.44	−0.59	0.50	−1.57	0.38
	Natal Country—Lithuania	11.74	249.67	−477.6	501.1	12.94	315.37	−605.17	631.06
	Natal Country—Norway	0.81	0.37	0.08	1.53	0.82	0.37	0.09	1.54
	Natal Country—Sweden	−0.11	0.35	−0.80	0.57	−0.11	0.35	−0.79	0.57
	Natal Country—UK	2.17	0.48	1.24	3.10	2.09	0.46	1.19	3.00
	Mixture 2	2.78	0.05	2.70	2.87	2.80	0.05	2.71	2.89
	Colourband—Finnish: *tsm*	0	0	0	0	−0.11	0.03	−0.16	−0.06
	Colourband—Spanish: *tsm*	0	0	0	0	−0.19	0.02	−0.21	−0.11
	Colourband—ELSA: *tsm*	0	0	0	0	−0.14	0.03	−0.19	−0.09

### 3.3 Sex‐Specific Survival

**FIGURE 3:**
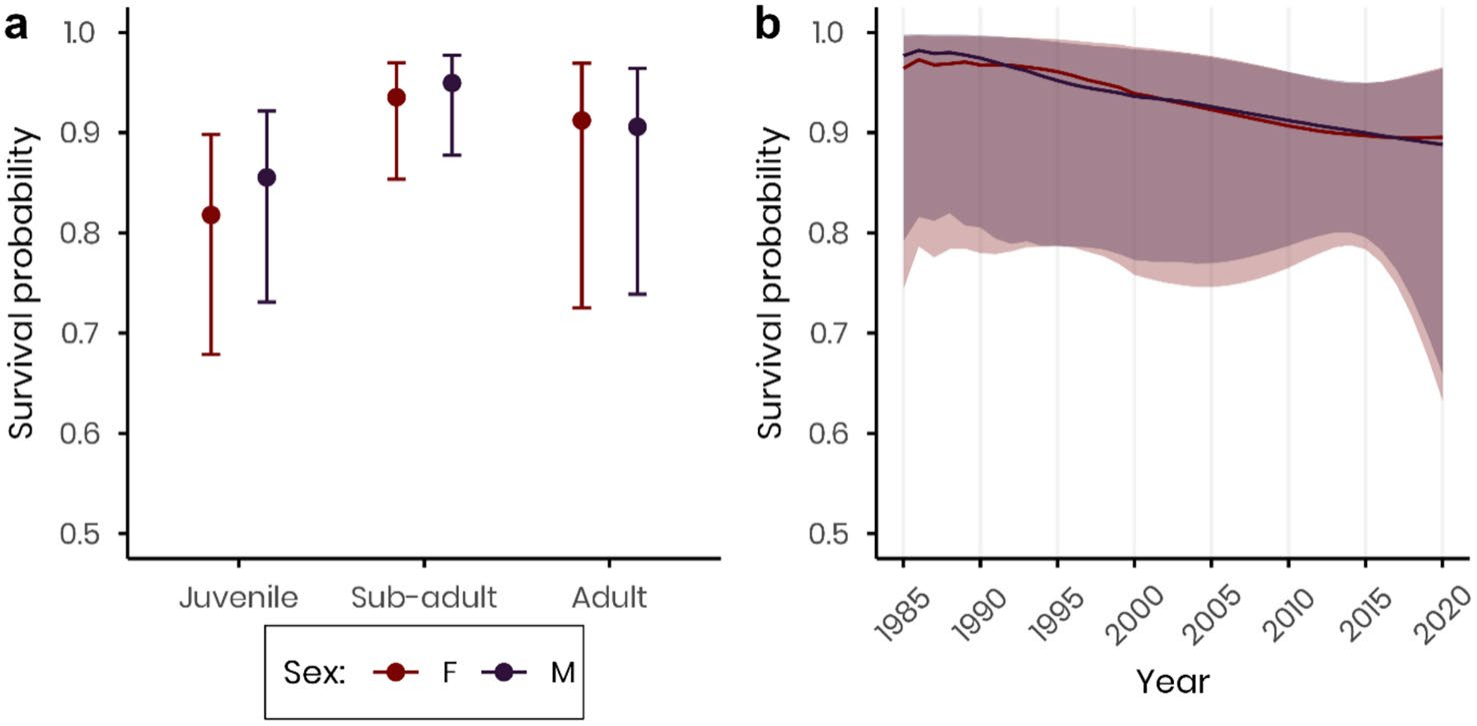
Sex‐specific survival probabilities of banded European cranes, between 1985 and 2021, (a) average per age‐class, and (b) over years, derived by model averaging. The colours represent the two different sexes, red: Females, and purple: Males. Solid lines or points represent the estimated values, while the shaded areas or error bars indicate the respective 95% confidence limits.

In this subset analysis, female and male cranes did not show significantly different survival probabilities across life stages (Figure 3, and Table 2). However, males showed slightly higher, though not significant, survival estimates during early stages (juvenile: 0.86 [0.73, 0.92] and sub‐adult: 0.95 [0.88, 0.98]) compared to females (respectively: 0.82 [0.68, 0.90] and 0.94 [0.85, 0.97]) (Figure 3a). Over time, the predicted survival trends for both sexes did not differ significantly (Figure 3b).

**TABLE 2:** Parameter beta estimates of model 1 and 2, including sex for estimating survival and detection probability of banded European cranes between 1985 and 2021. Key variables include sex, age classes: Juveniles (intercept), sub‐adults, adults; time since marking (tsm) in years, temporal trend (*T*), natal country and band types (colourband), for parameters (Par.) of mixture (pi), survival (Phi) and detection (p). LCI and UCI respectively represent lower and upper 95% confidence intervals.

Par.	Variable	Model 1				Model 2			
		Est.	SE	LCI	UCI	Est.	SE	LCI	UCI
pi	Intercept	−0.49	0.09	−0.66	−0.33	−0.52	0.09	−0.68	−0.35
Phi	Intercept	3.29	0.35	2.61	3.97	3.29	0.35	2.61	3.97
	Age	−0.23	0.14	−0.5	0.04	−0.23	0.14	−0.50	0.04
	Subadult	1.12	0.59	−0.05	2.28	1.11	0.59	−0.05	2.28
	Adult	1.89	1.15	−0.36	4.15	1.80	1.15	−0.45	4.05
	*T*	−0.08	0.01	−0.10	−0.05	−0.08	0.01	−0.10	−0.05
	Sex—M	0.43	0.51	−0.57	1.42	0.45	0.51	−0.54	1.45
	Age: *T*	0.01	0.01	−0.002	0.02	0.01	0.01	−0.002	0.02
	Subadult: *T*	0.01	0.02	−0.03	0.05	0.01	0.02	−0.03	0.05
	Adult: *T*	−0.02	0.04	−0.09	0.06	−0.01	0.04	−0.09	0.07
	Age: Sex—M	0.19	0.18	−0.16	0.54	0.16	0.18	−0.18	0.51
	Subadult: Sex—M	−0.20	0.87	−1.90	1.51	−0.19	0.87	−1.9	1.52
	Adult: Sex—M	−2.59	1.48	−5.49	0.32	−2.50	1.47	−5.39	0.39
	*T*: Sex—M	−0.01	0.02	−0.04	0.03	−0.01	0.02	−0.04	0.03
	Age: *T*: Sex—M	−0.01	0.01	−0.02	0.01	−0.01	0.01	−0.02	0.01
	Subadult: *T*: Sex—M	0.01	0.03	−0.05	0.07	0.01	0.03	−0.05	0.07
	Adult: *T*: Sex—M	0.08	0.05	−0.02	0.18	0.08	0.05	−0.02	0.18
p	Intercept	0.01	0.62	−1.21	1.22	−0.77	0.75	−2.23	0.69
	Colourband—Finnish	0.50	0.33	−0.15	1.15	0.32	0.47	−0.6	1.24
	Colourband—Spanish	−1.26	0.29	−1.83	−0.69	−0.47	0.51	−1.47	0.53
	Colourband—ELSA	−1.25	0.33	−1.89	−0.61	−0.74	0.53	−1.77	0.29
	*tsm*	−0.23	0.01	−0.25	−0.21	−0.18	0.04	−0.26	−0.11
	*T*	0.10	0.01	0.08	0.13	0.12	0.01	0.10	0.14
	Natal Country—Estonia	−0.32	0.58	−1.46	0.82	−0.43	0.58	−1.57	0.72
	Natal Country—Finland	−2.79	0.60	−3.97	−1.61	−2.77	0.62	−4.0	−1.55
	Natal Country—Germany	0.47	0.55	−0.61	1.55	0.39	0.55	−0.7	1.47
	Natal Country—Latvia	−1.38	0.73	−2.80	0.05	−1.57	0.72	−2.99	−0.15
	Natal Country—Lithuania	11.89	412.34	−796.3	820.1	11.75	407.95	−787.83	811.32
	Natal Country—Norway	2.46	0.66	1.17	3.75	2.43	0.66	1.13	3.73
	Natal Country—Sweden	0.08	0.56	−1.01	1.16	−0.01	0.56	−1.11	1.08
	Natal Country—UK	2.16	0.70	0.78	3.53	2.01	0.71	0.63	3.40
	mixture 2	3.08	0.08	2.92	3.24	3.11	0.08	2.94	3.27
	Colourband—Finnish: *tsm*	0	0	0	0	0.02	0.04	−0.07	0.10
	Colourband—Spanish: *tsm*	0	0	0	0	−0.08	0.04	−0.16	−0.01
	Colourband—ELSA: *tsm*	0	0	0	0	−0.07	0.04	−0.14	0.01

### 3.4 Cumulative Survival and Maximum Lifespan

**FIGURE 4:**
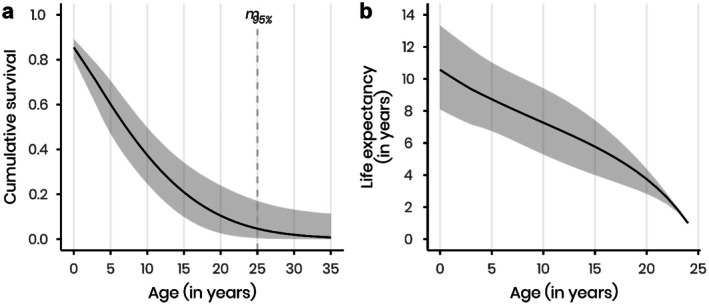
Cumulative survival and life expectancy of cranes. (a) Cumulative survival probabilities. The dashed vertical line marks the age at which cumulative survival probability drops below 5% (maximum lifespan m_95%_: 25 years). (b) Life expectancy at different ages. Solid lines represent the estimated values, while the shaded areas indicate the respective 95% confidence limits. All estimates are based on model averaging.

The cumulative survival probabilities showed a decline in survival, stabilising after 25 years old. Cumulative survival reached below 5% at 25 years, indicating a maximum lifespan (m_95%_) of 25 years for this population (Figure 4a).

### 3.5 Life Expectancy

The life expectancy decreased with age, as expected (Figure 4b). Life expectancy at birth was estimated at 10.5 years.

## 4 Discussion

Our study provides a comprehensive analysis of the survival rates of cranes ringed as juveniles across 11 different countries between 1985 and 2021. The results revealed lower survival rates in juveniles compared to adults, which is consistent with the higher vulnerability typically observed in younger age groups across bird species. In non‐juvenile cranes, average survival probability was highest in sub‐adult individuals, probably due to the absence of reproductive costs in that age class (Nur 1990; Stearns 1989; Williams 1966), and decreased progressively with increasing age, reflecting senescence (Promislow 1991). Part of the apparent decline in survival at older ages may be influenced by age‐related band deterioration; however, the databases do not systematically record partial or complete band loss, making this effect impossible to quantify. Annual survival rates showed different dynamics for each age class. Juvenile cranes experienced a clear and important decline in annual survival over the study period. Sub‐adults also showed a decline but less pronounced than that of juveniles. In contrast, adult cranes maintained a relatively constant survival rate over the years. Additionally, a subset of the data with individuals of known sex showed no substantial differences in survival between sexes, either across age classes or over time. The analysis of cumulative survival and life expectancy showed a substantial decline in survival beyond a certain age, indicating a limited maximum lifespan within this population. The life expectancy at birth of 10.5 years is considerably lower than the maximum lifespan observed in this population, which is of 25 years old. The large difference in longevity and maximum lifespan emphasises the high early mortality during juvenile and sub‐adult stages, but also that some cranes have the potential to live longer lives.

Our average survival estimates (juveniles: 0.85; sub‐adults: 0–89; and adults: 0.94) are similar to values obtained in previous studies on various crane species: the Eurasian crane, based on ring recoveries and resightings from 1989 to 2011 (0.85; Bautista and Alonso 2018); a Eurasian crane population reintroduced in the UK, from artificial rearing of young birds, and that is non‐migratory (survival probability range: juveniles 0.84–0.92, adults > 2 years old 0.89–0.95; Donaldson et al. 2023); Sandhill cranes (*Antigone canadensis*), with estimates ranging from 0.82 to 0.98 depending on life‐stages, sex and studied populations (Arnold et al. 2016; Bennett and Bennett 1990; Fronczak et al. 2015; Wheeler et al. 2019); Red‐crowned cranes ( *Grus japonensis* ; range: 0.80–0.91; Momose 2013); Whooping cranes ( *Grus americana* ), in which annual survival rates ranged from 0.72 to 1, depending on age, but also if the population was natural or reintroduced (Gil‐Weir et al. 2012; Link et al. 2003; Servanty et al. 2014). Several ecological and environmental factors may underlie the lower survival of juvenile and sub‐adult cranes compared with adults. Multiple mortality causes affect juvenile and immature individuals more than adults, but the decline in survival of non‐adult cranes over the last decades and the variation in response observed across age‐classes are difficult to explain by these drivers. For instance, juveniles face a higher predation risk, as they are not able to fly until they are around 10 weeks old (Johnsgard 1983), but most of this predation occurs before they are tagged. Most juveniles fledged soon after banding, even though walking remains their main transportation mean for many weeks after fledging. They then remain with their parents over the whole non‐breeding season until after the return migration from their winter quarters to their breeding areas (Alonso et al. 1984), which surely minimised their mortality. Another factor that may have contributed to higher first‐year mortality is the first migration failure. Indeed, juvenile birds have no experience in flying long distances and must rely on following their parents or other adults; despite this, they may suffer more from the consequences of such a long journey. For example, in White storks ( *Ciconia ciconia* ), the lower survival rate of juveniles was attributed to a higher energy cost, which is not well compensated by resting or foraging combined with lower flying skills (Rotics et al. 2016). In Golden eagles ( *Aquila chrysaetos* ), the energy cost of flying was also shown to be decreasing with age (Nourani et al. 2024). However, these mortality causes cannot explain the negative trend observed in non‐adult survival over the study period.

A probable cause of that decline in juvenile and sub‐adult survival may have been habitat degradation over the last four decades, driven either by decreasing water levels in wetlands as a consequence of climate change, or by agriculture expansion and urban development. Increased climatic variability over the last decades, with alternating floods and droughts, has impacted water levels and resource availability for cranes (Hansbauer et al. 2014). These negative factors may have affected cranes in their breeding areas, but also in their staging and wintering areas. Climate change is also modifying the timing of migration, with cranes arriving earlier at the spring staging sites, and also at the autumn staging or wintering areas (Filippi‐Codaccioni et al. 2011; Lundgren 2012; Orellana‐Macías et al. 2020; Salvi 2016a; Végvári 2015). These phenological shifts may reflect conditions of food shortages, drying wetlands (Orellana‐Macías et al. 2020), or other threats at staging and wintering sites, posing long‐term risks to cranes (Végvári 2015). These effects have also been detected in other species. For example, a study of Song sparrow ( *Melospiza melodia* ) in California found that warmer and drier winters projected by 2100 could significantly increase adult survival but slightly decrease juvenile survival. The differences found in that study likely arise as adult survival benefits from milder winters, while juvenile survival depends more on food availability, which is indirectly improved by increasing rainfall in the preceding season (Dybala et al. 2013). Therefore, similar climatic conditions can have differential effects on different life stages (Fay et al. 2017), highlighting the need to understand age‐specific responses to environmental variability for accurate demographic predictions. Our findings also align with a recent study on white storks, which demonstrated that juvenile survival exhibits higher sensitivity to climate change, as well as other environmental and human‐induced changes (Martín et al. 2021).

Another factor that may have affected juvenile cranes more than adults is the intensive drainage of peatlands and wetlands which has reduced suitable habitats essential for breeding, staging, and wintering, altering their distribution and habitat use (Leito et al. 2005), and potentially affecting the survival of the most vulnerable age classes or specific stage (e.g., moulting). Moreover, as waterbodies dry out, juveniles and flightless moulting adult cranes become more vulnerable to predators, as lower water levels allow easier access for predatory mammals, as shown in marsh bird studies (Schmidt et al. 2023). However, adult moult is typically lasting 5 weeks, and their size makes adults less vulnerable than juveniles. In addition, changing agricultural practices, such as monoculture expansion and increased pesticide use in the last decades, also pose serious risks and may have contributed to differential mortality rates among age classes. Pesticides can harm birds directly by poisoning them when foraging on agricultural fields, or indirectly by reducing food availability (Hemminger et al. 2022; Moreau et al. 2022). Juveniles depend more on invertebrates during their first months of life, while adults can subsist on plant matter (Nowald and Fleckstein 2001). In a study from northeast Germany, invertebrate availability varied significantly across crane territories, with the frequency of beetles found in faecal samples correlating with observed frequency in pitfall traps (Nowald and Fleckstein 2001). This shows that crane families may adapt their diet to the availability of these prey, and suggests that a shortage of invertebrates could result in a less healthy diet for juvenile cranes. Since pesticide use and water drainage can diminish the availability of invertebrates and amphibians (Austin 2018), juveniles might suffer from pesticides more than adults (Outhwaite et al. 2022).

Beyond these environmental changes, increased population densities at the breeding areas represent additional pressures on the birds. The increase in population size observed over the last decades in Europe (Alonso et al. 2016; Leito et al. 2008; Prange and Ilyashenko 2019) has allowed cranes to expand their breeding range towards north, south, and west (Prange and Ilyashenko 2019). In addition, the population increase has also led to a higher density of nesting pairs in most regions (Leito et al. 2003; Prange and Ilyashenko 2019; Tichackova and Lumpe 2014). As a result, breeding pairs are forced to use suboptimal or low‐quality habitats which may affect their hatching success and juvenile survival (Barwisch et al. 2022; Nowald et al. 2010). A study on the Wandering albatross ( *Diomedea exulans* ) showed that survival of young birds was primarily affected by population density, while recruitment age was more strongly affected by long‐term climate conditions (Fay et al. 2015, 2017). Also, higher density at wintering or staging sites could increase disturbance, making it more difficult for juveniles to access food compared to more dominant adults (Alonso et al. 1997), and risking juvenile‐parents separation. Finally, dense populations favour the spread of diseases, like avian influenza, which can cause significant mortality and probably affect juvenile birds more than adults because of their underdeveloped immune system (Miller 2022). For instance, an avian influenza (AIV‐H5N1) outbreak in the Hula Valley in Israel, the largest wintering site of this species in the Middle East, led to significant mortality of 10,000 cranes in 2021 (Farnoushi et al. 2023).

Regarding the sex‐specific survival, our results showed no difference in survival between sexes across age classes. Although sex was treated as a fixed attribute due to limited information on when and how it was determined, any potential bias is likely minimal as both sexes are identified at similar ages. Those results are consistent with findings in another crane species, the Whooping crane, in which authors found little difference in the estimated survival of males and females (Servanty et al. 2014). Eurasian cranes, as other species of cranes, do show weak sex dimorphism in terms of body mass and size (e.g., tarsus, wing, or head length; Swengel 1992, Alonso et al. 2019). Both sexes participate practically equally in parental care, with males primarily focusing on territorial defence against predators and neighbouring territory holders, while females spend more time caring for their offspring and feed at a slightly slower rate than males. This can be interpreted as a cost of maternal care that would compensate for the higher mortality risks of males resulting from territorial or family defence (Alonso et al. 2004; Nowald 2001). In the Black‐necked crane ( *Grus nigricollis* ), during incubation, both nest attendance by males and females was not different (Zhang et al. 2017). Our results suggest that both roles entail overall similar survival costs or that selection has balanced survival outcomes across these differing parental strategies.

With its broad spatio‐temporal scope, leveraging citizen science data, this study provides a foundation for addressing challenges faced by this crane population. Although our results showing declining survival in juveniles and sub‐adults are worth attention, they do not necessarily indicate an imminent population decline. In fact, increases in population size and juvenile mortality have occurred simultaneously over the last four decades, with no detrimental effects on the population trend so far. However, our study does warn that habitat deterioration caused by humans and climate change has probably been affecting juvenile survival for decades. If these effects were to affect adult survival or productivity in the future, they would be really detrimental to the species. This is because, as in other long‐lived species, crane demography is more sensitive to adult survival and productivity than to juvenile survival. Previous Eurasian crane demographic models indicated that significant declines in productivity (50%), small declines in adult survival (10%), or a combined 20% drop in productivity and 10% in adult survival could lead to population extinction (J. A. Alonso et al. 1991; Alonso, Quintanilla, and López 1986). In order to update these models now that we have reliable survival values for the Eurasian crane, it is important to obtain productivity data for this species, the other crucial parameter for population trends. In the meantime, it is urgent to start implementing conservation measures immediately, before habitat deterioration significantly affects the survival or reproductive rates of adults. Among these measures, we highlight the following: First, to avoid urban and agricultural development or other habitat changes that reduce food resources needed by crane pairs to raise their offspring (invertebrates and small vertebrates). In this regard, it is particularly important to maintain an adequate water table, avoiding any human action that leads to the drying up of ponds, bogs or sedge meadows. These measures should not only be applied in breeding areas but also in staging and wintering areas, where the negative consequences seem to be currently affecting mainly juveniles but could also extend their impact to adults in the future. Second, to prevent the indiscriminate use of pollutants and pesticides in all main crane areas. Finally, infrastructures such as power lines and wind turbines should not be installed in areas important for cranes, since they represent a major mortality cause in this species. Future research that could inform these conservation actions include investigating in detail all mortality causes and environmental pressures, including those derived from climate change and induced by human impacts.

## Author Contributions

**Morgane Gicquel:** data curation (equal), formal analysis (lead), visualization (lead), writing – original draft (lead), writing – review and editing (lead). **Juan C. Alonso:** conceptualization (equal), data curation (equal), funding acquisition (equal), investigation (equal), supervision (equal), writing – review and editing (supporting). **Lovisa Nilsson:** conceptualization (equal), funding acquisition (equal), investigation (equal), supervision (equal), writing – review and editing (supporting). **Matthew Low:** resources (equal), supervision (equal), writing – review and editing (supporting). **Javier A. Alonso:** investigation (equal), writing – review and editing (supporting). **Dmitrijs Boiko:** investigation (equal), writing – review and editing (supporting). **Damon Bridge:** investigation (equal), writing – review and editing (supporting). **Patrick Dulau:** investigation (equal), writing – review and editing (supporting). **Thomas Heinicke:** investigation (equal), writing – review and editing (supporting). **Anne Kettner:** investigation (equal). **Yosef Kiat:** investigation (equal), writing – review and editing (supporting). **Petras Kurlavičius:** investigation (equal), writing – review and editing (supporting). **Sigvard Lundgren:** investigation (equal), writing – review and editing (supporting). **Michael Modrow:** investigation (equal). **Günter Nowald:** investigation (equal), writing – review and editing (supporting). **Ivar Ojaste:** investigation (equal), writing – review and editing (supporting). **Alain Salvi:** investigation (equal), writing – review and editing (supporting). **Jostein Sandvik:** investigation (equal). **Markéta Ticháčková:** investigation (equal), writing – review and editing (supporting). **Antonio Torrijo:** investigation (equal), writing – review and editing (supporting). **Jari Valkama:** investigation (equal), writing – review and editing (supporting). **Zsolt Végvári:** investigation (equal), writing – review and editing (supporting). **Johan Månsson:** conceptualization (equal), funding acquisition (equal), investigation (equal), resources (equal), supervision (equal), writing – review and editing (supporting).

## Funding

This work was supported by Norddeutsche Stiftung für Umwelt und Entwicklung, Kranichschutz Deutschland/NABU, Eesti Maaülikool, PM170161PKMH, Carl Tryggers Stiftelse för Vetenskaplig Forskning, CTS:297, Ministerio de Educación y Formación Profesional, PB87‐0389, PB91‐0081, Naturvårdsverket.

## Conflicts of Interest

The authors declare no conflicts of interest.

## Supporting information


**Appendix S1:** Supporting Information.

**Appendix S2:** Supporting Information.

**Appendix S3:** Supporting Information.

## Data Availability

Capture history data are uploaded as Supporting Information. The raw datasets supporting the findings of this study are not publicly available. However, they may be available upon request from the European Crane Working Group participants, subject to approval and any necessary confidentiality agreements.
