# Supplementary material for: Age‐Specific Survival Estimation of a Eurasian Crane Population Highlights a Long‐Term Decline in Juvenile Survival
Source: Ecol Evol. 2026 Feb 3;16(2):e72779. doi: 10.1002/ece3.72779 (PMC12865728; doi:10.1002/ece3.72779)
Supplement: Supplementary file 3 — Appendix S3: Supporting Information. [file ECE3-16-e72779-s001.docx]

# Supporting information

## Appendix A. Data information

**Table A.1:** Information linked to natal countries of banded cranes: number of cranes banded originating from each country, number of resightings from the cranes originating from each country, number of cranes never resighted after banding and respective proportion.

| **Natal country** | **Number of individuals** | **Number of resightings** | **Start of banding scheme** | **Maximum time since marking (in years)** | **Number of individuals never resighted** | **Proportion never resighted** |
| --- | --- | --- | --- | --- | --- | --- |
| Sweden | 822 | 25,190 | 1985 | 36 | 151 | 18% |
| Finland | 998 | 8,091 | 1987 | 34 | 206 | 21% |
| Germany | 2,417 | 121,582 | 1988 | 33 | 496 | 21% |
| Norway | 137 | 6,934 | 1996 | 25 | 15 | 11% |
| Estonia | 430 | 6,958 | 1997 | 24 | 91 | 21% |
| Czech Republic | 61 | 1,493 | 2002 | 19 | 15 | 25% |
| United Kingdom* | 88 | 750 | 2010 | 11 | 0 | 0% |
| Latvia | 89 | 1,466 | 2012 | 9 | 21 | 24% |
| Lithuania | 7 | 261 | 2016 | 5 | 0 | 0% |

* Reintroduced population, using eggs collected in Germany and hatched and hand-reared before release (see Donaldson *et al.* 2023).

**Table A.2:** Number of cranes banded with information on types and extent of band types use.

| **Band type** | **Number of individuals** | **Year of first use** | **Maximum time since marking (in years)** |
| --- | --- | --- | --- |
| Alphanumeric | 161 | 1985 | 36 |
| Spanish | 800 | 1988 | 33 |
| Finnish | 744 | 1990 | 31 |
| ELSA | 3,386 | 2001 | 20 |

## Appendix B. Goodness of fit

Table B.1: Results of the goodness-of-fit tests performed with the R package *r2ucare* on the whole dataset. Pearson’s χ^2^ is the test statistic, df are the degrees of freedom. Test 2.CT is the test component for trap dependence, Test 3.SR is the test component for transience, and the other two components indicate overdispersion.

|  | **Pearson's χ^2^** | **df** | **P-value** | **Sign-test** |
| --- | --- | --- | --- | --- |
| Test 2.CL | 443.74 | 174 | < 0.001 |  |
| Test 2.CT | 4,944.77 | 32 | < 0.001 | -59.18 |
| Test 3.SM | 148.64 | 103 | 0.002 |  |
| Test 3.SR | 328.09 | 33 | < 0.001 | 6.65 |

## Appendix C. Best models from full dataset used in model averaging

**Table C.1:** Top CJS mixture candidate models used for model averaging. The models were sorted by QAICc from low to high. Model formula, number of parameters (npar), QAICc, ΔQAICc, QAkaike weight (w), and QDeviance (QDev.) are displayed. Full model list is available in previous publication (Gicquel et al., 2025).

| **Phi** | **p** | **pi** | **npar** | **QAICc** | Δ**QAICc** | **w** | **QDev.** |
| --- | --- | --- | --- | --- | --- | --- | --- |
| ~(Age + Subadult + Adult) * *T* | ~colourband + *tsm* + *T* + natal country + mixture | ~1 | 24 | 4,941.19 | 0.00 | 0.55 | 2,214.12 |
| ~(Age + Subadult + Adult) * *T* | ~colourband * *tsm* + *T* + natal country + mixture | ~1 | 27 | 4,941.58 | 0.39 | 0.45 | 2,208.50 |

## Appendix D. Best model using median ĉ correction results

We alternatively used the median ĉ (ĉ = 1.45) value to correct our models ranking and compare with the best models obtained from using Fletcher ĉ correction (c-hat = 8.57 ; see Appendix C). With the median ĉ, only one model was selected as the best, others having a ΔQAICc > 7. The difference with the best ranking models selected with Fletcher ĉ correction, is that natal country is also considered for it’s effect on survival probability (Table D.1).

Results are similar. Beta estimates are within the same order (Table D.2), and predictions are almost identical. For instance, juveniles have an average survival of 0.87 95%CI[0.83,0.90], sub-adults of 0.94 95%CI[0.93,0.96] and adults of 0.90 95%CI[0.87,0.92] (Figure D.1).

Regarding the effect of natal country, cranes survival varies between 0.88 to 0.95 depending on the natal origin of the individual (Figure D.3).

**Table D.1:** Top CJS mixture candidate model. Model formula, number of parameters (npar), QAICc, ΔQAICc, QAkaike weight (w), and QDeviance (QDev.) are displayed.

| **Phi** | **p** | **pi** | **npar** | **QAICc** | Δ**QAICc** | **w** | **QDev.** |
| --- | --- | --- | --- | --- | --- | --- | --- |
| ~(Age + Subadult + Adult) * *T* + natal country | ~colourband * *tsm* + *T* + natal country + mixture | ~1 | 35 | 28,942.91 | 0.00 | 1 | 13,025.53 |

**Table D.2:** Parameter beta estimates of the best fitting model using median c-hat correction (see Table D.1) for estimating survival and detection probability of banded cranes between 1985 and 2021. Key variables include age classes: Age (juvenile intercept), sub-adults, adults; time since marking (tsm) in years, temporal trend (T), natal country and band types (colourband), for parameters (Par.) of mixture (pi), survival (Phi) and detection (p). LCI and UCI respectively represent lower and upper 95% confidence intervals.

| Par. | Variable | Estimate | SE | LCI | UCI |
| --- | --- | --- | --- | --- | --- |
| pi | Intercept | -0.18 | 0.06 | -0.29 | -0.07 |
| Phi | Intercept | 3.72 | 0.31 | 3.12 | 4.32 |
|  | Age | -0.13 | 0.05 | -0.23 | -0.02 |
|  | Subadult | -0.01 | 0.33 | -0.65 | 0.63 |
|  | Adult | -1.22 | 0.47 | -2.15 | -0.29 |
|  | *T* | -0.09 | 0.01 | -0.10 | -0.07 |
|  | Age : *T* | 0.003 | 0.002 | -0.001 | 0.01 |
|  | Subadult : *T* | 0.04 | 0.01 | 0.02 | 0.07 |
|  | Adult : *T* | 0.09 | 0.02 | 0.06 | 0.12 |
|  | Natal Country - Estonia | 0.54 | 0.24 | 0.08 | 1.01 |
|  | Natal Country - Finland | 0.25 | 0.23 | -0.19 | 0.7 |
|  | Natal Country - Germany | 0.04 | 0.22 | -0.39 | 0.47 |
|  | Natal Country - Latvia | 0.48 | 0.31 | -0.16 | 1.05 |
|  | Natal Country - Lithuania | 1.38 | 1.063 | -0.7 | 3.46 |
|  | Natal Country - Norway | 0.43 | 0.25 | -0.06 | 0.92 |
|  | Natal Country - Sweden | 0.29 | 0.23 | -0.15 | 0.73 |
|  | Natal Country - United Kingdom | 0.16 | 0.29 | -0.4 | 0.72 |
| p | Intercept | -2.97 | 0.41 | -3.78 | -2.16 |
|  | Colourband - Finnish | 2.25 | 0.23 | 1.8 | 2.7 |
|  | Colourband - Spanish | 1.90 | 0.23 | 1.44 | 2.35 |
|  | Colourband - ELSA | 1.76 | 0.25 | 1.27 | 2.24 |
|  | *tsm* | -0.08 | 0.03 | -0.13 | -0.03 |
|  | *T* | 0.10 | 0.01 | 0.08 | 0.11 |
|  | Natal Country - Estonia | -1.58 | 0.36 | -2.29 | -0.87 |
|  | Natal Country - Finland | -1.72 | 0.37 | -2.44 | -1.0 |
|  | Natal Country - Germany | 0.41 | 0.36 | -0.29 | 1.11 |
|  | Natal Country - Latvia | -0.77 | 0.54 | -1.82 | 0.28 |
|  | Natal Country - Lithuania | 12.80 | 540.64 | -1046 | 1072 |
|  | Natal Country - Norway | 0.75 | 0.38 | 0.01 | 1.49 |
|  | Natal Country - Sweden | -0.19 | 0.35 | -0.89 | 0.52 |
|  | Natal Country - United Kingdom | 2.08 | 0.51 | 1.08 | 3.08 |
|  | mixture 2 | 2.82 | 0.05 | 2.73 | 2.91 |
|  | Colourband – Finnish : *tsm* | -0.11 | 0.03 | -0.16 | -0.06 |
|  | Colourband – Spanish : *tsm* | -0.15 | 0.03 | -0.20 | -0.11 |
|  | Colourband – ELSA : *tsm* | -0.14 | 0.03 | -0.18 | -0.09 |

**
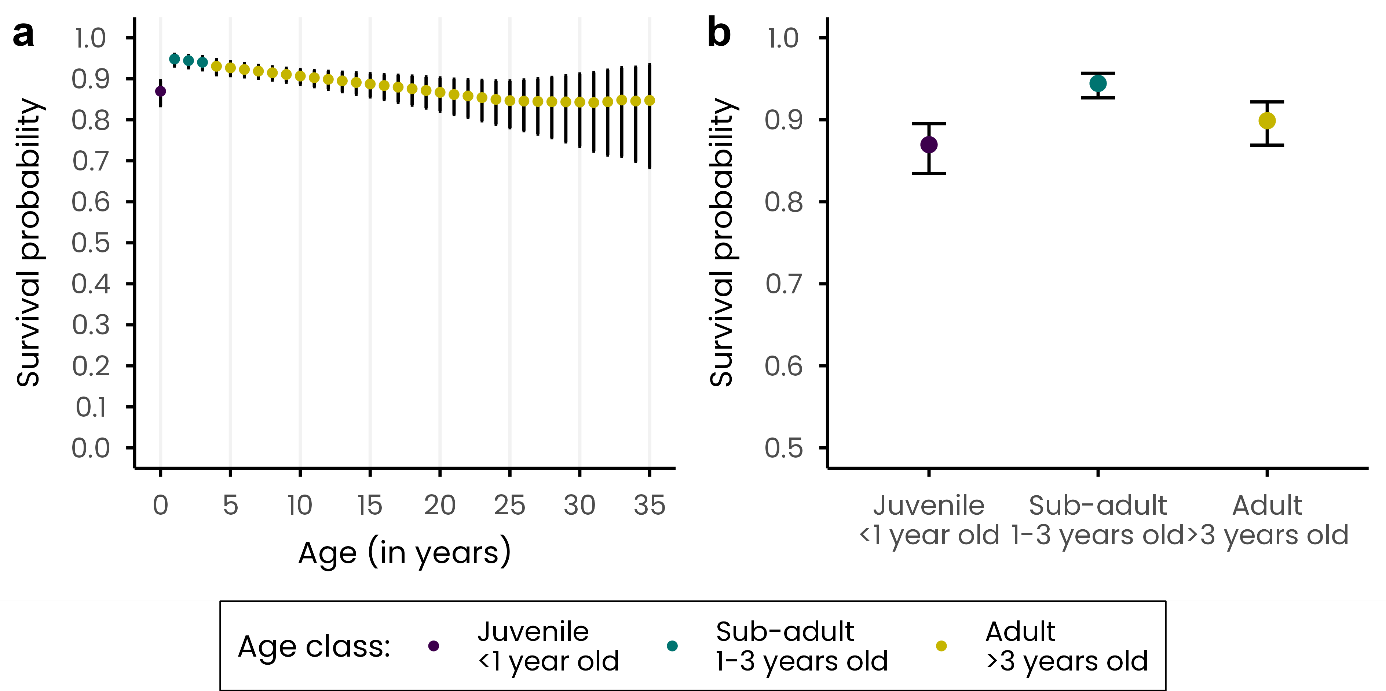
**

**Figure D.1:** Survival probabilities of banded European cranes, between 1985 and 2021. (a) Age-specific survival, and (b) average survival probability per age-class. The colours represent three different age classes, purple: juveniles (less than 1 year old), green: sub-adults (1-3 years old) and yellow: adults (older than 3 years old).

**
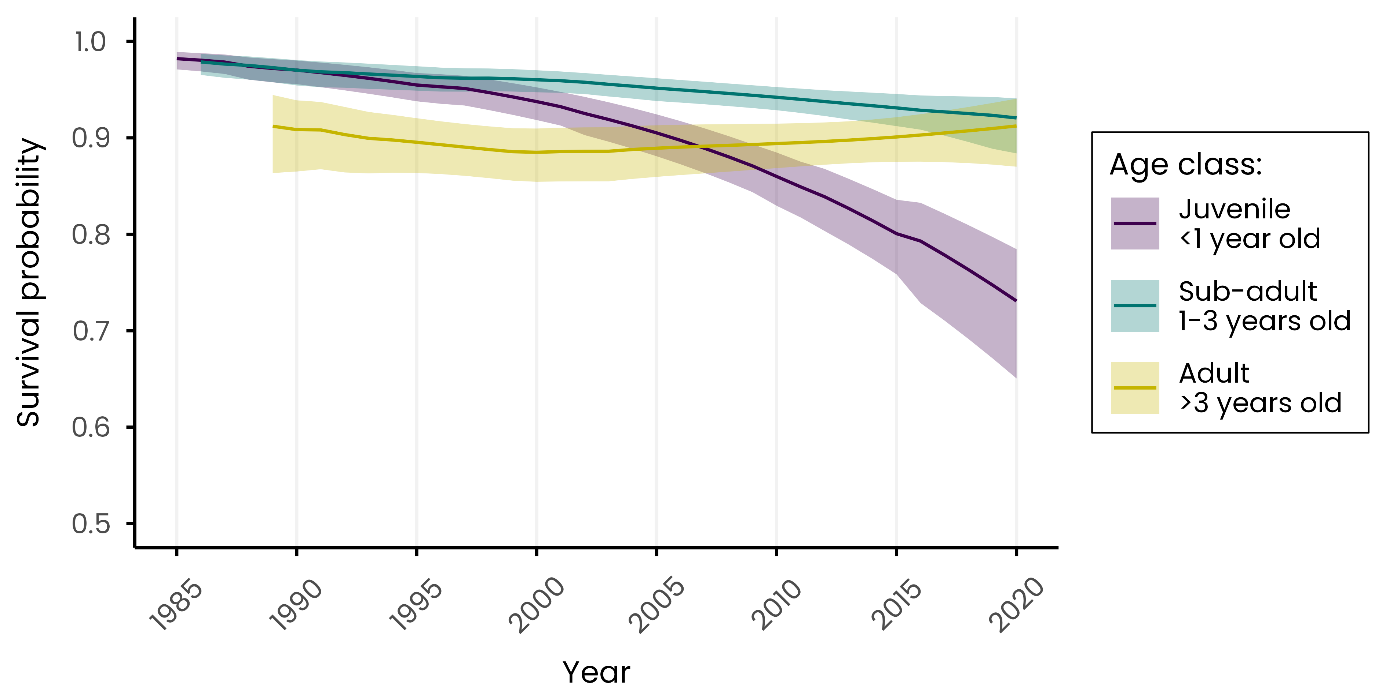
**

**Figure D.2:** Survival probabilities of banded European cranes for specific age-class over the years (temporal trend *T*), between 1985 and 2021. The colours represent three different age stages, purple: juveniles (less than 1 year old), green: sub-adults (1-3 years old) and yellow: adults (older than 3 years old).


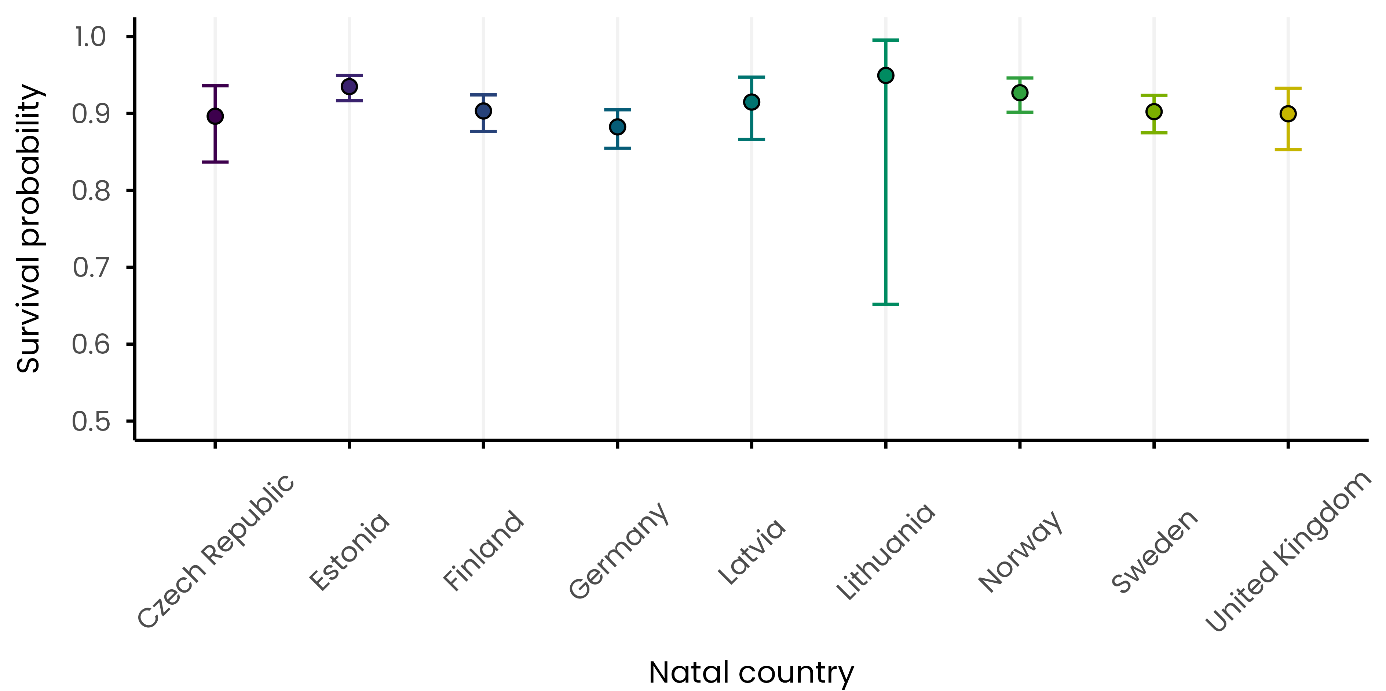


**Figure D.3:** Survival probabilities of banded European cranes for specific natal country origin, between 1985 and 2021.

## Appendix E. Best models from data subset including sex effect used in model averaging

**Table E.1:** Top CJS mixture candidate models including sex effect used for model averaging. The models were sorted by QAICc from low to high. Model number, formula, number of parameters (npar), QAICc, ΔQAICc, QAkaike weight (w), and QDeviance (QDev.) are displayed.

| **Phi** | **p** | **pi** | **npar** | **QAICc** | Δ**QAICc** | **w** | **QDev.** |
| --- | --- | --- | --- | --- | --- | --- | --- |
| ~(Juvenile + Subadult + Adult) * T * Sex | ~colourband + tsm + T + natal country + mixture | ~1 | 32 | 1,740.95 | 0.00 | 0.87 | 904.23 |
| ~(Juvenile + Subadult + Adult) * T * Sex | ~colourband * tsm + T + natal country + mixture | ~1 | 35 | 1,744.75 | 3.80 | 0.13 | 902.00 |
